# A Darwinian approach to the development of the vascular system in the vertebrates

**DOI:** 10.1007/s10238-025-01633-y

**Published:** 2025-03-27

**Authors:** Domenico Ribatti

**Affiliations:** Department of Translational Biomedicine and Neuroscience, University of Bari Medical School, Piazza Giulio Cesare, 11, 70125 Bari, Italy

**Keywords:** Blood vessels, Endothelium, Invertebrates, Pericytes, Vascular system, Vertebrates

## Abstract

The vascular system originated around 600 million years ago. Endothelial cells evolved between 540 and 510 million years ago, and endothelial heterogeneity also developed. In invertebrates, two typologies have been described, the so-called open and closed systems, whereas in vertebrates only a closed system is present. In mammals, the presence of smooth muscle cells in the walls of small arteries regulates blood pressure and distribution to different organs; capillaries are involved in the exchange of gasses and metabolites; veins return the blood to the heart, whereas lymphatic vessels collect interstitial fluids and white blood cells and are in continuity with the venous system. Endothelial heterogeneity is the consequence of the different interactions of endothelium with the organ and tissue microenvironment including stromal cells, which is mediated by soluble factors or cell–cell/cell–extracellular matrix interactions leading to a particular phenotype of the endothelium. In this context, the heterogeneity of endothelial cells reflects specific responses to different microenvironments and their specialization to perform different functions, leading to different subsets of endothelial cells with unique gene expression patterns.

## Open and closed circulatory systems

The vascular system originated around 600 million years ago. Endothelial cells evolved between 540 and 510 million years ago, and endothelial heterogeneity also developed [22]. In invertebrates, two typologies have been described, the so-called open and closed systems, whereas in vertebrates only a closed system is present. In the open circulatory system, occurring in arthropods (e.g., insects and crustaceans) and non-cephalopod mollusks (e.g., clams, snails, and slugs), the blood called hemolymph mixed with the interstitial fluid, pumps into the body cavity called hemocoel surrounding the different organs, allowing gas and nutrient exchanges [20], and then returns in the ventricle (arthropods) or via the atrium (mollusks) through openings called ostia [7]. The hemocoel is bordered not by mesoderm-derived mesothelial cells, but rather by the basal surface of the tissue cells themselves. Tissues are in direct contact with the hemolymph during gas and nutrient exchanges. In these animals, the mesoderm forms coelomic cavities during embryogenesis. However, the cavities and their cell lining regress in adults. Some populations of mesodermal cells reaggregate and form blood vessels (e.g., dorsal vessels in insects).

The closed circulatory system is present in different invertebrates including annelids, cephalopods (e.g., octopus squid), and non-vertebrate chordates, as well as in vertebrates. In closed systems, the blood remains inside distinct channels, where it is physically separated from the intercellular fluid, body cells, and coelom. The blood contained inside blood vessels moves unidirectionally from the heart to the different organs and then returns to the heart again. Blood does not directly contact tissues during gas and nutrient exchanges. Birds, having a closed circulatory system, are thought to have moved more agilely, allowing them to obtain food faster and possibly to prey on the insects [32]. In vertebrates, different closed circulatory systems are recognizable. In detail, fish have two-chambered hearts with unidirectional circulation; amphibians and most non-avian reptiles have three-chambered hearts with double circulation; mammals and birds have four-chambered hearts with double circulation.

In mammals, the presence of smooth muscle cells in the walls of small arteries regulates blood pressure and distribution in different organs; capillaries are involved in the exchange of gasses and metabolites; veins return the blood to the heart, whereas lymphatic vessels collect interstitial fluids and white blood cells and are in continuity with the venous system.

## The evolution of endothelium

In most invertebrates, the luminal side of the vascular channels is not covered by cells, but it is lined by a mesodermal matrix [25, 28]. Some invertebrates have an incomplete lining of endothelial-like cells of mesothelial/epithelial or myoepithelial origin [25, 39]. The more appropriate term for this cell type is an amebocyte, representing a type of circulating hemocyte, which may or not be an evolutionary precursor of the endothelial cell. Amebocytes are not connected by intercellular junctions typical of vertebrate endothelial cells [21].

An endothelial cell is defined as one covering the luminal space of the vessels, forming a continuous layer having a basal luminal polarity with the apical surface facing the lumen [22]. Specialized junctional complexes keep these cells adherent to each other [25]. The endothelial cells are not a passive mechanical barrier but can selectively regulate the transit of molecules and water, and control coagulation, contractility, inflammation, and immune response [24]. Endothelial cells are also the first line of contact with blood flowing in the lumen. In this context, the role of flow in regulating endothelial cell morphology is relevant. Changes in shear stress induce the polarization of endothelial cells, which involves changes in cell orientation in the direction of blood flow [4]. Fluid shear stress also triggers activation of specific transcription factors [43].

Primitive endothelium and blood cells share a common progenitor called the hemangioblast [40] that are related to ancient blood‐derived cells called amebocytes [25, 31]. Hemangioblast originates from the mesoderm, mainly in areas closer to the endoderm and in the aorta–gonad–mesonephros region. Direct evidence of the existence of hemangioblasts has been reported [8, 26]. These cells are also thought to be the precursors of thrombocytes, suggesting a close relationship between endothelial cells and thrombocytes [19].

Vasculogenesis, i.e., the formation of blood vessels starting from mesodermal precursors namely angioblasts, leads to the formation of the first major intra-embryonic blood vessels, such as the dorsal aorta and the cardinal veins, and the formation of the primary vascular plexus in the yolk sac [33]. The isolation of putative bone marrow-derived endothelial progenitor cells (EPCs) has stimulated research into the nature of these cells and their use for regenerative medicine applications [3].

Angiogenesis is defined as a new blood vessel sprouting from preexisting vessels. This can be accomplished through endothelial sprouting or non-sprouting (intussusceptive) microvascular growth (IMG) [33, 34]. This latter postulated that the capillary network increases its complexity and vascular surface by the insertion of a multitude of transcapillary pillars, a process called “intussusception” [10]. After forming a primitive vascular plexus, remodeling transforms the plexus into an organized network of arteries, capillaries, and veins, establishing directional flow, and the association with pericytes and smooth muscle cells.

In pathological conditions, such as tumor growth, non-angiogenic growth in the absence of angiogenesis may occur through alternative mechanisms of vascularization, namely vascular co-option, in which cancer cells exploit preexisting vessels, or vasculogenic mimicry, in which the cancer cells form vascular channels [14, 35].

There is a common number of molecules expressed both on mesodermal and endothelial cells, including CD44, prostaglandin E2, angiotensin-converting enzyme, plasminogen activator inhibitor, and acetylate low-density lipoprotein [25]. Adhesion molecules forming intercellular junctions also characterize endothelial cells, regulating vascular permeability in different organs [9]. Adherent junctions are common to all endothelial cells, whereas tight and gap junctions graduate within different vascular segments [41].

Main proteins investigated to understand endothelial evolution are vascular endothelial growth factor (VEGF), its receptor (VEGFR), von Willebrand factor (vWF), and vascular cell adhesion molecule 1 (VCAM1) [16, 18, 25]. Various splice isoforms of VEGF-A (VEGF-A 189, VEGF-A 165, VEGF-A 144, VEGF-A 121), other ligand members in the family (VEGF-B, VEGF-C, platelet-derived growth factor, PDGF, fibroblast growth factor-2, FGF-2, placental growth factor, PlGF), and primary receptors, such as VEGFR-1, VEGFR-2, and co-receptors Neuropilin-1 and -2, have been discovered. These molecules and their overlapping expression patterns in the developing and adult vasculature highlighted the complexity of the vascular system. Other signaling systems closely related with vertebrate angiogenesis have not been identified in invertebrate vascular growth. Angiopoietins regulate vascular stability in vertebrates. This family includes four proteins (Angiopoietin-1–4), which are ligands of two tyrosine kinase receptors (Tie1 and Tie2). Ang-1 activates Tie2 signaling promoting vessel assembly and stabilization by facilitating recruitment and high association with mural cells and mediating survival signals for endothelial cells [13].

## Pericytes

The pericytes evolve from myoepithelial cells, as has been noted in different models, e.g., the avian embryonal aorta, where the mesothelial cells are subject to epithelial-mesenchymal transition and then can go on to express actin and become muscle cells located inside the arterial wall [30]. Zimmermann first described the pericytes in 1923 [42], showing that these cells are present around capillaries in a wide range of species, are continuous with smooth muscle cells of arteries and veins, and that their contraction controls capillary permeability. Tissues differ widely in their number of pericytes or endothelial coverage.

Capillaries are associated with pericytes, a multipotent contractile cell type on the abluminal surface of the endothelial cells, responsible for the secretion of a tissue-specific basal lamina. The term pericyte has expanded over time to encompass the description of any resident periendothelial cell. There are both molecular and phenotypic features that are either distinct to, or in common between, the following cell types: brain capillary pericytes, arterial, venous, or lymphatic periendothelial smooth muscle cells, kidney podocytes, coronary vascular pericytes, liver stellate cells, myofibroblasts, dental pulp stem cells [2]. Many of the specific cell types retain mesenchymal multipotency into adulthood in multiple species. It is therefore essential to define the cell type with both molecular markers. For example, the large transmembrane glycoprotein encoded by the chondroitin sulfate proteoglycan 4 (CSPG4) gene, also known as nerve/glia antigen 2 (NG2), a surface proteoglycan expressed throughout all stages of pericyte development, is also expressed by many multipotent cell types, such as mesenchymal, keratinocyte, and radial glia stem cells [5, 6]. The multifunctional nature of pericytes and their involvement in a complex signaling pathway make them essential in the homeostasis of the vascular system.

## Endothelial heterogeneity

The hagfish display already has heterogeneity anatomical, functional, and molecular of its endothelial cells [1, 12, 31, 45]. Several studies suggest that endothelial cells are not genetically committed to an arterial or venous phenotype but are plastic and adapt to the expression of the arterial or venous-specific genes based on local environmental cues [23, 27].

Heterogeneity is the consequence of the different interactions of endothelium with the organ and tissue microenvironment including stromal cells, which is mediated by soluble factors or cell–cell interactions leading to a particular phenotype of the endothelium. In this context, the heterogeneity of endothelial cells reflects specific responses to different microenvironments and their specialization to perform different functions, leading to different subsets of endothelial cells with unique gene expression patterns [13].

Endothelial cells from distinct vascular beds are different and contain subsets of cells expressing unique gene expression patterns. For example, 15 distinct cell subtypes have been identified in mouse brain endothelial cells, and 17 distinct cell types have been identified in mouse lung endothelial cells [15]. Further insights gained from scRNA studies [17] show that capillary endothelial cells in a tissue are characterized by the greatest heterogeneity in gene expression pattern compared to arterial, venous, or lymphatic endothelial cells.

Organ-specific endothelial cells may actively shape the local tissue microenvironment by releasing various paracrine/juxtacrine factors, namely “angiocrine factors” [31], as it has been demonstrated in different organs, including the liver, pancreas, brain, lung, heart, kidney, skin, bone marrow [36–38] as well as in pathological conditions, including cancer [29].

## Concluding remarks

As the geneticist and evolutionary biologist Theodosius Dobzhansky asserted, “Nothing in biology makes sense except in the light of evolution” [11]. Therefore, all life science innovation in the coming decades will rely to a significant degree on approaches now being developed by leaders in evolutionary medicine. Williams and Nesse’s pioneering 1991 review [44] remarked on vulnerability to disease as the product of evolutionary processes underscoring the importance of historical, functional, and contextual perspectives for medicine. Darwinian medicine is the application of modern evolutionary theory to human health and illness, lying at the crossroads between theoretical and applied with human biology, medical anthropology, psychology, and physiology. It begins examining the overall concept of evolutionary medicine, advancing through a series of topics showing the scope of impact that evolutionary theory has on medicine today. This approach has permitted important advances in the understanding of different pathological conditions, including cancer and autoimmune diseases, as well as in organogenesis, and the case of the vascular system, as we have summarized in this article. The vascular system is an example of how evolutionary analyses of development reveal that new anatomical structures often arise by co-opting existing structures and molecular pathways that were established earlier in the history of life.

## Data Availability

No datasets were generated or analyzed during the current study.

## References

[1] Aird WC. Endothelial cell heterogeneity. Cold Spring Harb Perspect Med. 2012;2: a006429. 22315715 10.1101/cshperspect.a006429 PMC3253027

[2] Armulik A, Genové G, Betsholtz C. Pericytes: developmental, physiological, and pathological perspectives, problems, and promises. Dev Cell. 2011;21:193–215. 21839917 10.1016/j.devcel.2011.07.001

[3] Asahara T, Murohara T, Sullivan A, Silver M, van der Zee R, Li T, et al. Isolation of putative progenitor endothelial cells for angiogenesis. Science. 1997;275:964–7. 9020076 10.1126/science.275.5302.964

[4] Barakat AI. Blood flow and arterial endothelial dysfunction: Mechanisms and implications. Comptes Rendus Phys. 2013;14:479–96.

[5] Birbrair A, Zhang T, Wang ZM, Messi ML, Mintz A, Del Bono O. Pericytes at the intersection between tissue regeneration and pathology. Clin Sci. 2015;128:81–93. 10.1042/CS20140278 PMC4200531 25236972

[6] Birbrair, A. (ed). Pericyte biology. Novel concepts. Adv Exp Med Biol 1109, Springer, 2018. 10.1007/978-3-030-02601-1_1 30523585

[7] Bourne G, Redmond JR, Jorgensen DD. Dynamics of the Mollusca circulatory system: open versus closed. Physiol Zool. 1990;63:140–66.

[8] Choi K, Kennedy M, Kazarov A, Papadimitriou JC, Keller G. A common precursor for hematopoietic and endothelial cells. Development. 1998;125:725–32. 9435292 10.1242/dev.125.4.725

[9] Dejana E. Endothelial cell-cell junctions: happy together. Nat Rev Mol Cell Biol. 2004;5:261–70. 15071551 10.1038/nrm1357

[10] Djonov VG, Galli AB, Burri PH. Intussusceptive arborization contributes to vascular tree formation in the chick chorioallantoic membrane. Anat Embryol. 2000;202:347–57. 10.1007/s004290000126 11089926

[11] Dobzhansky T. Nothing in biology makes sense except in the light of evolution. Am Biol Teacher. 1973;35:125–9.

[12] Douglas Coffin J. The evolution of comparative biology of vascular development and the endothelium. In: Aird WC, editor. Endothelial Biomedicine. Cambridge: Cambridge Univeristy Press; 2007. p. 50–8.

[13] Fagiani E, Christofori G. Angiopoietin in angiogenesis. Cancer Lett. 2013;328:18–26. 22922303 10.1016/j.canlet.2012.08.018

[14] Harris AL, Kerr DJ, Pezzella F, Ribatti D. Accessing the vasculature in cancer: revising an old hallmark. Trends Cancer. 2024;10:1038–51. 39358088 10.1016/j.trecan.2024.08.003

[15] He L, Vanlandewijck M, Mae MA, Andrae J, Ando K, Del Gaudio F, et al. Single-cell RNA sequencing of mouse brain and lung vascular and vessel associated cell types. Sci Data. 2018;5: 180160. 30129931 10.1038/sdata.2018.160 PMC6103262

[16] Holmes DI, Zachary I. The vascular endothelial growth factor (VEGF) family: Angiogenic factors in health and disease. Genome Biol. 2005;6:209. 15693956 10.1186/gb-2005-6-2-209 PMC551528

[17] Kalucka J, de Rooij L, Goveia J, Rohlenova K, Dumas SJ, Meta E, et al. Single-cell transcriptome atlas of murine endothelial cells. Cell. 2020;180:764–79. 32059779 10.1016/j.cell.2020.01.015

[18] Lenting PJ, Pegon JN, Groot E, de Groot PG. Regulation of von Willebrand factor-platelet interactions. Thromb Haemost. 2010;104:449–55. 20539912 10.1160/TH09-11-0777

[19] Levin, J. (2019). The evolution of mammalian platelets. In Platelets (4th ed., pp. 1–23). Academic Press.

[20] Majna JN. Structure, function, and evolution of the gas exchangers: comparative perspectives. J Anat. 2002;128:81–93. 10.1046/j.1469-7580.2002.00099.x PMC1570919 12430953

[21] Moller PC, Philpott CW. The circulatory system of Amphioxus (Branchiostoma floridae). I. Morphology of the major vessels ofthe pharyngeal area. J Morphol. 1973;139:389–406. 4708448 10.1002/jmor.1051390403

[22] Monahan-Earley R, Dvorak AM, Aird WC. Evolutionary origins of the blood vascular system and endothelium. J Thromb Haemost. 2013;11(Suppl 1):46–66. 23809110 10.1111/jth.12253 PMC5378490

[23] Moyon D, Pardanaud L, Li Y, Bréant C, Eichmann A. Plasticity of endothelial cells during arterial-venous differentiation in the avian embryo. Development. 2001;128:3359–3570. 10.1242/dev.128.17.3359 11546752

[24] Munoz-Chapuli R, Perez-Pomares JM. Cardiogenesis: an embryological perspective. J Cardiovasc Transl Res. 2010;3:37–48. 10.1007/s12265-009-9146-1 20560033

[25] Munoz-Chapuli R, Carmona R, Guadix JA, Macias D, Perez-Pomares JM. The origin of the endothelial cells: an evo-devo approach for the invertebrate/vertebrate transit ion of the circulatory system. Evol Dev. 2005;7:351–8. 15982372 10.1111/j.1525-142X.2005.05040.x

[26] Nishikawa SI, Nishikawa S, Hirashima M, Matsuyoshi N, Kodama H. Progressive lineage analysis by cell sorting and culture identifies FLK1+VE-cadherin^+^ cells at a diverging point of endothelial and hemopoietic lineages. Development. 1998;125:1747–57. 10.1242/dev.125.9.1747 9521912

[27] Othman-Hassan K, Patel K, Papoutsi M, Rodriguez-Niedenführ M, Christ B, Wilting J. Arterial identity of endothelial cells is controlled by local cues. Dev Biol. 2001;237:398–409. 10.1006/dbio.2001.0383 11543623

[28] Pascual-Amaya J, Albuixech-Crespo B, Somorjai IML, Carmona R, Oisi Y, Alavarez S, et al. The evolutionary origins of chordate hematopoiesis and vertebrate endothelia. Dev Biol. 2013;375:182–92. 23201012 10.1016/j.ydbio.2012.11.015

[29] Pasquier J, Ghiabi P, Chouchane L, Razzouk K, Rafii S, Rafii A. Angiocrine endothelium: from physiology to cancer. J Transl Med. 2020;18:52. 32014047 10.1186/s12967-020-02244-9 PMC6998193

[30] Perez-Pomares JM, Macias-Lopez D, Garcia-Garrido L, Munoz-Chapuli R. Immunohistochemical evidence for a mesothelial contribution to the ventral wall of the avian aorta. Histochem J. 1999;31:771–9. 10661320 10.1023/a:1003997919311

[31] Rafii S, Butler JM, Ding BS. Angiocrine functions of organ-specific endothelial cells. Nature. 2016;529:316–25. 26791722 10.1038/nature17040 PMC4878406

[32] Reiber CL, Mcgaw IJ. A review of the “Open” and “Closed” circulatory systems: new terminology for complex invertebrate circulatory systems in light of current findings. Int J Zool. 2009. 10.1155/2009/301284.

[33] Ribatti D. Genetic and epigenetic mechanisms in the early development of the vascular system. J Anat. 2006;208:139–52. 16441559 10.1111/j.1469-7580.2006.00522.x PMC2100191

[34] Ribatti D, Crivellato E. Sprouting angiogenesis, a reappraisal. Dev Biol. 2012;372:157–65. 23031691 10.1016/j.ydbio.2012.09.018

[35] Ribatti D, Pezzella F. Overview on the different patterns of tumor vascularization. Cells. 2021;10:639. 33805699 10.3390/cells10030639 PMC8000806

[36] Ribatti D. Liver angiocrine factors. Tissue Cell. 2023a;81:102027. 10.1016/j.tice.2023.102027. 36657255 10.1016/j.tice.2023.102027

[37] Ribatti D, d’Amati A. Bone angiocrine factors. Front Cell Dev Biol. 2023;11:1244372. 37601109 10.3389/fcell.2023.1244372 PMC10435078

[38] Ribatti D, Ligresti G, Nicosia RF. Kidney endothelial cell heterogeneity angiocrine activity and paracrine regulatory mechanisms. Vascul Pharmacol. 2023;148:107139. 36539108 10.1016/j.vph.2022.107139 PMC10828957

[39] Ruppert EE, Travis PB. Hemoglobin-containing cells of neodasys (gastrotricha, chaetonotida). I Morphology and Ultrastructure J Morphol. 1983;175:57–64. 30060640 10.1002/jmor.1051750106

[40] Sabin F. Studies on the origin of blood vessels and of red corpuscles as seen in the living blastoderm of the chick during the second day of incubation. Contribut Embryol. 1920;9:213–62.

[41] Simionescu M, Simionescu N, Palade E. Segmental differentiation of cell junctions in the vascular endothelium. J Cell Biol. 1975;67:863–85. 1202025 10.1083/jcb.67.3.863 PMC2111645

[42] Zimmermann KW. Der feinere Bau der Blutkapillaren. Z Anat Entwick. 1923;68:29–36.

[43] Wang W, Ha CH, Jhun BS, Wong C, Jain MK, Jin ZG. Fluid shear stress stimulates phosphorylation-dependent nuclear export of HDAC5 and mediates expression of KLF2 and eNOS. Blood. 2010;115:2971–9. 20042720 10.1182/blood-2009-05-224824 PMC2854437

[44] Williams GC, Nesse RM. The dawn of Darwinian medicine. Q Rev Biol. 1991;66:1–22. 2052670 10.1086/417048

[45] Yano K, Gale D, Massberg S, Cheruvu PK, Monahan-Earley R, Morgan ES, et al. Phenotypic heterogeneity is an evolutionarily conserved feature of the endothelium. Blood. 2007;109:613–5. 16990601 10.1182/blood-2006-05-026401

